# Contribution of TRPC Channels to Intracellular Ca^2 +^ Dyshomeostasis in Smooth Muscle From mdx Mice

**DOI:** 10.3389/fphys.2020.00126

**Published:** 2020-02-20

**Authors:** Jose R. Lopez, Arkady Uryash, Gilles Faury, Eric Estève, Jose A. Adams

**Affiliations:** ^1^Department of Research, Mount Sinai Medical Center, Miami, FL, United States; ^2^Department of Neonatology, Mount Sinai Medical Center, Miami, FL, United States; ^3^HP2, CHU Grenoble Alpes, Inserm, University Grenoble Alpes, Grenoble, France

**Keywords:** Duchenne, calcium, TRPC, smooth muscle, mdx

## Abstract

Duchenne muscular dystrophy (DMD) is an irreversible muscle disease characterized by a progressive loss of muscle function, decreased ambulation, and ultimately death as a result of cardiac or respiratory failure. DMD is caused by the lack of dystrophin, a protein that is important for membrane stability and signaling in excitable cells. Although vascular smooth muscle cells (VSMCs) dysfunction occurs in many pathological conditions, little is known about vascular smooth muscle function in DMD. We have previously shown that striated muscle cells, as well as neurons isolated from dystrophic (mdx) mice have higher intracellular Ca^2+^ ([Ca^2+^]_i_) and Na^+^ ([Na^+^]_i_) concentrations and decreased cell viability in comparison with wild type (Wt). Experiments were carried out in isolated VSMCs from mdx (a murine model of DMD) and congenic C57BL/10SnJ Wt mice. We found elevated [Ca^2+^]_i_ and [Na^+^]_i_ in VSMCs from mdx mice compared to Wt. Exposure to 1-oleoyl-2-acetyl-sn-glycerol (OAG), a TRPC3 and TRPC6 channel activator, induced a greater elevation of [Ca^2+^]_i_ and [Na^+^]_i_ in mdx than Wt VSMCs. The OAG induced increases in [Ca^2+^]_i_ could be abolished by either removal of extracellular Ca^2+^ or by SAR7334, a blocker of TRPC3 and TRPC 6 channels in both genotypes. Mdx and Wt VSMCs were susceptible to muscle cell stretch-induced elevations of [Ca^2+^]_i_ and [Na^+^]_i_ which was completely inhibited by GsMTx-4, a mechanosensitive ion channel inhibitor. Western blots showed a significant upregulation of TRPC1 -3, -6 proteins in mdx VSMCs compare to age-matched Wt. The lack of dystrophin in mdx VSMCs produced a profound alteration of [Ca^2+^]_i_ and [Na^+^]_i_ homeostasis that appears to be mediated by TRPC channels. Moreover, we have been able to demonstrate pharmacologically that the enhanced stretch-induced elevation of intracellular [Ca^2+^] and concomitant cell damage in mdx VSMCs also appears to be mediated through TRPC1, -3 and -6 channel activation.

## Introduction

Duchenne muscular dystrophy (DMD) is an X-linked inherited neuromuscular disorder caused by mutations in the dystrophin gene (Hoffman et al., 1987). Dystrophin is localized in the plasmalemma of excitable cells connecting the cytoskeleton of the cell to the extracellular matrix (Ibraghimov-Beskrovnaya et al., 1992; Waite et al., 2012). While initial clinical manifestations of DMD are related to skeletal muscle weakness (Chakkalakal et al., 2005), the development of dilated cardiomyopathy occurs occasioning heart failure and death (Nigro et al., 1990) and a non-progressive cognitive impairment has been observed in DMD patients (Bresolin et al., 1994; Anderson et al., 2002). Disturbance of intracellular [Ca^2+^] has been reported in skeletal muscle from DMD patients (Lopez et al., 1987) and mdx mice (Turner et al., 1988; Altamirano et al., 2013, 2014), in cardiac cells from mdx mice (Mijares et al., 2014) and in cortical and hippocampal neurons isolated from mdx mice (Lopez et al., 2016).

Although X-linked neuromuscular pathologies have been extensively studied in striated muscle, the implications of lack of dystrophin in smooth muscle in patients with DMD and mdx mice have not been adequately studied. Studies on mdx dystrophic gastric and intestinal smooth muscle cells have revealed impaired nitrergic relaxation and an increase in spontaneous tone, which have been attributed to the impairment of intracellular Ca^2+^ homeostasis (Mule et al., 1999; Baccari et al., 2000; Mule and Serio, 2001). In addition, in DMD patients, dysfunction in small airways (Begin et al., 1980), constipation (Nowak et al., 1982), gastric dilatation, intestinal pseudo-obstructions and acute gastric dilatation (Leon et al., 1986; Barohn et al., 1988; Jaffe et al., 1990) have been described. Furthermore, endothelial cell damage, platelet adhesion, and aggregation in small blood vessels have been observed in DMD patients (Miike et al., 1987). However, the functional implications of the lack of dystrophin in vascular smooth muscle cells (VSMCs) are mostly unknown.

In the current study, we show for the first time that dystrophin deficiency of VSMCs leads to dysfunctional regulation of [Ca^2+^]_i_ and [Na^+^]_*i*,_ which appears to be mediated by an influx of these ions through the transient receptor potential canonical (TRPC) channels. Furthermore, mechanical stretch elicited a further elevation of [Ca^2+^]_i_ in VSMCs from mdx compared to Wt, which was inhibited by the removal of extracellular calcium and by TRPC channel blockade.

## Materials and Methods

### Animal Model

Wt (C57BL/10SnJ) and mdx (CS7BL/10ScSn-mdx) male (6 months old) mice were obtained from breeding colonies at the Mount Sinai Medical Center, from founders initially obtained from the Jackson Laboratory (Bar Harbor, ME). All protocols used in the study were performed following the recommendations in the Guide for the Care and Use of Laboratory Animals of the National Institutes of Health and approved by the institution (IACUC Protocol #19090).

### Primary Culture of VSMC

Mdx and their Wt non-dystrophic littermates were euthanized using CO_2_ or cervical dislocation. VSMCs were isolated using a modification of a previously described method (Ray et al., 2001). In brief, the aorta was dissected from its origin at the left ventricle to the iliac bifurcation, and the vessel was cut and placed in Hank’s Balanced Salt Solution (HBSS) (Thermo Fisher Scientific, FL, United States). Using a dissecting microscope, the fat tissue and the adventitia were removed; then, the aorta was irrigated with HBSS plus 2.5 μg/mL Fungizone (Thermo Fisher Scientific, FL, United States). The vessel was open longitudinally, and with sterile cotton swabbed, the endothelial layer of cells was gently removed and then cut into small segments (around 4 mm^2^ each). The aorta segments were incubated in HBSS containing 1 mg/ml collagenase type 2 (Worthington Biochemical Corporation, NJ, United States) for 30 min. Then, the solution containing collagenase type 2 was replaced with a solution containing 1 mg/ml collagenase type 2 and 0.5 mg/ml elastase (Worthington Biochemical Corporation, NJ, United States). After the second digestion step, the remaining tissue was mechanically dissociated in the dish by flushing through a series of decreasing size fire-polished pipettes. Fresh HBSS was then added to stop the enzymatic digestion, and the cell suspension was centrifugated at 600 × *g*. The cell pellet was resuspended and centrifugated again at 600 × *g* and then transferred to a Matrigel-coated 24-well cell culture plate containing smooth muscle cell growth medium (SGM-2, Lonza, GA, United States). Isolated VSMC were cultured in a humidified atmosphere (37°C) and for 7–10 days after platting before experimentation.

### Assessment of VSMC Functionality

The following criteria were used to judge the functionality of VSMCs: (i) no cell shortening was observed when they perfused with the Ca^2+^ containing Ringer solution (1.8 mM Ca^2+^) and (ii) they contracted in response to electrical stimuli (1 ms square pulse duration, ∼1.5 × threshold voltage).

### Measurements of Resting [Ca^2+^]_i_ and [Na^+^]_i_

Double-barreled Ca^2+^ and Na^+^ selective microelectrodes were prepared as described previously (Eltit et al., 2013). Single smooth muscle cells were impaled with either a Ca^2+^ – or Na^+^-selective double-barreled microelectrode, and their potentials were recorded via a high-impedance amplifier (WPI Duo 773 electrometer; World Precision Instruments, FL, United States). Criteria for successful impalement of single muscle cells included an (i) abrupt drop to a steady level of Vm more negative than −55 mV, (ii) a recording stable for both potentials (Vm and Ca potential) for at least 60 s and (iii) an quick return to baseline on the exit of the microelectrodes from the cell. The specific Ca^2 +^ potential (V_Cae_) or Na^+^ potential (V_Nae_) was obtained by subtracting the V_Ca_ potential or V_Na_ from the 3 M KCl microelectrode potential (Vm); Vm, and the specific Ca^2+^- Na^+^ potentials were stored in a computer for future analysis.

### Muscle Mechanical Stretch

VSMCs were seeded on flexible-bottomed culture plates coated with poly-L-lysine (Flexcell International Corp., NC, United States). After 48 h to allow for cell attachment and spreading, Wt and mdx VSMCs were bathing with Ringer solution and then subjected to mechanical stretch elongation of 30 cycles/min (0.5 Hz), 20% elongation using a Flexcell FX 5000 tension system for 5 min. After the cyclic stretch, to estimate cell damage, the medium was collected for the determination of lactate dehydrogenase (LDH) activity (released by VSMCs) using the LDH kit from Sigma-Aldrich (St. Louis, MO, United States) according to the manufacturer’s instructions. Parallel series of Flex culture plates not subjected to stretching served as controls. At the time of collection of the medium for LDH determination, [Ca^2+^]_i_ or [Na^+^]_i_ was measured in the stretched VSMCs using ion-selective microelectrodes.

### Western Blot Analysis

Mdx and Wt anesthetized mice were euthanized using CO_2_. The aorta was dissected as described above (Primary culture of VSMC). Aortic total protein extraction was performed using a modified Millipore enzyme buffer with added 0.5% Triton-X-100 for 1 h digestion and lysis step. After proteins transfer from the gel, we spliced the membrane horizontally according to the molecular weight of proteins of interest. Then, individual membrane strips were incubated with the primary anti-TRPC1, TRPC3, TRPC6, and dystrophin antibodies (Uryash et al., 2015). The corresponding protein size was determined based on the protein standard marker. The levels of target protein(s) were normalized to loading control using the housekeeper protein β-actin.

### Solutions

The normal Ringer’s solution contained the following (in mM): 135 NaCl, 5 KCl, 1.8 CaCl_2_, 1 MgCl_2_, 5 glucose, 3.6 NaHCO_3_ (pH 7.4). In all experiments, Wt and mdx VSMCs were perfused and equilibrated with the Ringer’s solution aerated with a mixture of 95% O_2_ and 5% CO_2_ at 37°C. For the Ca^2+^ free solution CaCl_2_ was omitted and 2 mM MgCl_2_ and 1 mM EGTA were added in its place. 1-oleoyl-2-acetyl-sn-glycerol (OAG) (100 μM) a TRPC3, and TRPC6 channel activator, SAR7334 (0.1 and 1 μM) a TRPC3 and TRPC6 channel blocker, GsMTx4 (5 μM) a specific mechanosensitive channel inhibitor, nifedipine (10 μM) a selective voltage-gated Ca^2+^ channel blocker solutions were prepared from stock solutions.

### Statistical Analysis

All values are reported as mean ± SD. Student’s *t*-test or analysis of variance (1-way ANOVA and Tukey’s *post hoc* tests were used to determine significance. *p* < 0.05 was considered statistically significant. *n*_mice_: number of mice used experimentally, *n*_cell_: represents the number of successful measurements carried out.

## Results

### [Ca^2+^]_i_ and [Na^+^]_i_ Dyshomeostasis in mdx Vascular Smooth Cells

**FIGURE 1:**
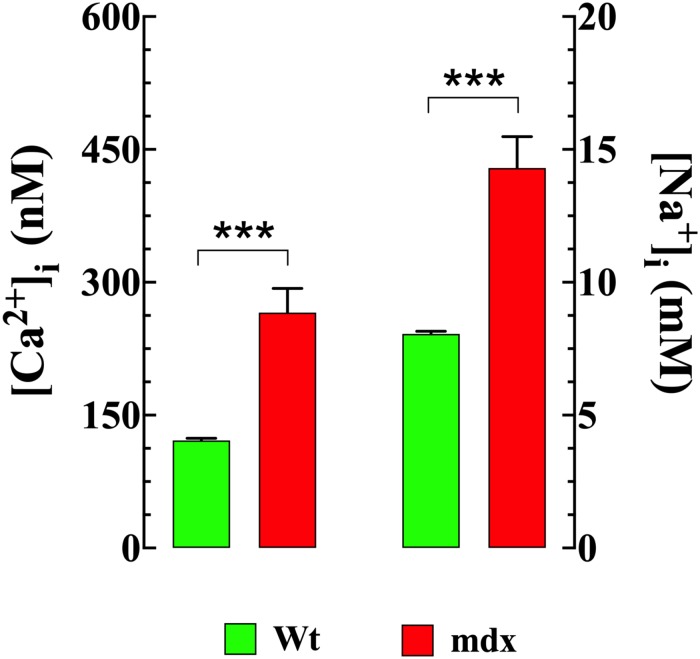
Elevated [Ca^2+^]_i_ and [Na^+^]_i_ in mdx VSMCs. [Ca^2+^]_i_ or [Na^+^]_i_ was measured in quiescent VSMCs isolated from Wt, and mdx mice using double-barreled ion-specific microelectrodes. [Ca^2+^]_i_ and [Na^+^]_i_ were significantly higher in mdx than Wt VSMCs. For [Ca^2+^]_i_ *measurements*: n_mice_ = 3/experimental condition, *n*_cell_ = 41–48/genotype; For [Na^+^]_i_ measurements: *n_mice_* = 3/experimental condition, *n*_cell_ = 25–27/genotype. Values are expressed as means ± S.D. Student’s *t*-test, ***p ≤ 0.001.

Striated muscle cells from DMD patients and mdx mice (Lopez et al., 1987; Altamirano et al., 2013, 2014; Mijares et al., 2014) show elevated intracellular Ca^2+^ and Na^+^. Therefore, intracellular [Ca^2+^] and [Na^+^] were measured in VSMCs isolated from Wt and mdx mice using double-barreled ion-specific microelectrodes. [Ca^2+^]_i_ was elevated in mdx VSMCs (266 ± 27 nM, *n* = 45) compared to that observed in Wt cells (121 ± 3 nM, *n* = 41), (*p* < 0.001) (Figure 1). Similarly, [Na^+^]_i_ was higher in mdx VSMCs (14 ± 1.2 mM *n* = 25) than observed in Wt cells (8 ± 0.1 mM *n* = 25) (*p* < 0.001) (Figure 1).

### OAG a TRPC3 and TRPC6 Activator, Induced Elevation of [Ca^2+^]_i_ and [Na^+^]_i_ in VSMCs

**FIGURE 2:**
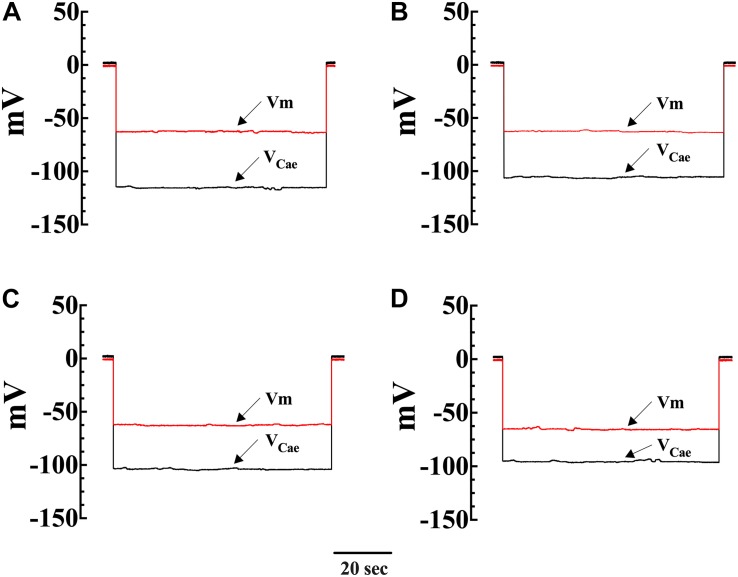
Simultaneous measurements of Vm and [Ca^2+^]_i_ in single smooth muscle cells from control Wt and mdx mice. Effects of OAG. Vm is the membrane potential recorded with a conventional microelectrode filled with 3 M KCI, and V_Cae_ is the potential recorded with the calcium-selective electrode after the subtraction of Vm. V_Cae_ potential represents cell intracellular [Ca^2+^]. In Wt, [Ca^2+^]_i_ was 124 nM, and the Vm was –62 mV before OAG treatment and represents the cell intracellular [Ca^2 +^ ] **(A)**. After incubation in OAG 100 μM [Ca^2+^]_i_ increased to 187 nM with no effect on Vm (–63 mV) **(B)**. The measurements of Vm and [Ca^2+^]_i_ before and after OAG treatment were carried in the same muscle cell. In the mdx muscle cell Vm: was –62 mV and [Ca^2+^]_i_ was 258 nM before OAG **(C)** and after OAG incubation, [Ca^2+^]_i_ rose to 492 nM after OAG, with no change in Vm (–65 mV) **(D)**. The determinations of Vm and [Ca^2+^]_i_ before and after OAG treatment were carried in two distinct muscle cells.

**FIGURE 3:**
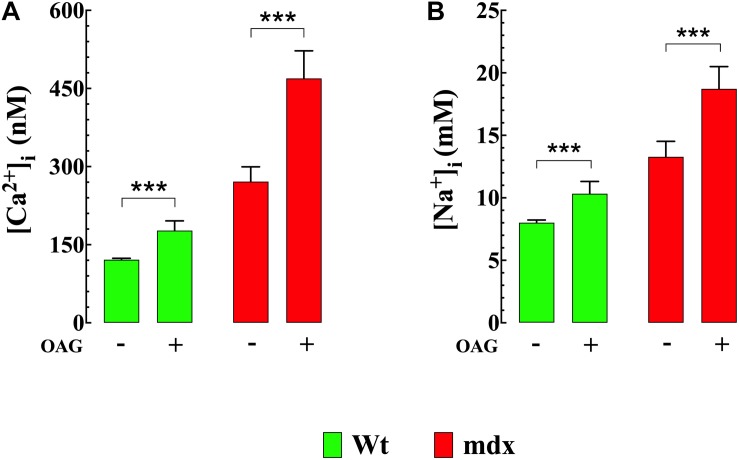
OAG induces elevation of [Ca^2+^]_i_ and [Na^+^]_i__._ Exposure of quiescent Wt and mdx VSMc to 1-oleoyl-2-acetyl-sn-glycerol (OAG) 100 μM induced a significant elevation of [Ca^2+^]_i_ **(A)** and [Na^+^]_i_ **(B)**, which were greater in mdx than Wt muscle cells. Over the horizontal axis are indicated the experimental conditions used to measure [Ca^2+^]_i_ and [Na^+^]_*i.*_ For [Ca^2+^]_i_ measurements: *n*_mice_ = 4/experimental condition, *n*_cell_ = 21–27/genotype. For [Na^+^]_i_ measurements: *n*_mice_ = 3/experimental condition, *n*_cell_ = 23–25/genotype. Values are expressed as means ± S.D. for each condition. Student’s *t*-test ***p ≤ 0.001.

To directly investigate the effect of diacylglycerol (DAG) on [Ca^2+^]_i_ and [Na^+^]_i_ in smooth muscle cells, VSMCs were exposed to the DAG analog 1-oleoyl-2-acetyl-sn-glycerol (OAG) which activates TRPC3 and TRPC6 channels (Hofmann et al., 1999). Incubation in OAG (100 μM) for 10 min produces an elevation of [Ca^2+^]_i_ and [Na^+^]_i_ in both genotypes. Figures 2A–D show representative measurements of the resting membrane potential (Vm) and [Ca^2+^]_i_ from Wt and mdx VSMCs before and after exposure to OAG. In Wt VSMCs OAG provoked an increase of [Ca^2+^]_i_ by 46%, from 121 ± 3 nM, *n* = 22, to 177 ± 19 nM, *n* = 25 (*p* < 0.001), and in mdx VSMCs by 73%, from 271 ± 29 nM, *n* = 25, to 470 ± 55 nM, *n* = 27 (*p* < 0.001) (Figure 3A). [Na^+^]_i_ was also significantly elevated in Wt by 25% and in mdx VSMC by 46% upon incubation in OAG (Figure 3B). We examined the possible involvement of the voltage-gated Ca^2+^ channels in the OAG-induced elevation of [Ca^2+^]_i_ by pretreating cultured VSMCs with nifedipine 10 μM, a specific voltage-gated Ca^2+^ channel blocker. Nifedipine had no significant effects on OAG-induced elevation of [Ca^2+^]_i_ in either Wt or mdx VSMCs (Supplementary Figure S1).

### Removal of Extracellular Ca^2+^ Reduced [Ca^2+^]_i_ and Prevented OAG-Induced Elevation of [Ca^2+^]_i_

**FIGURE 4:**
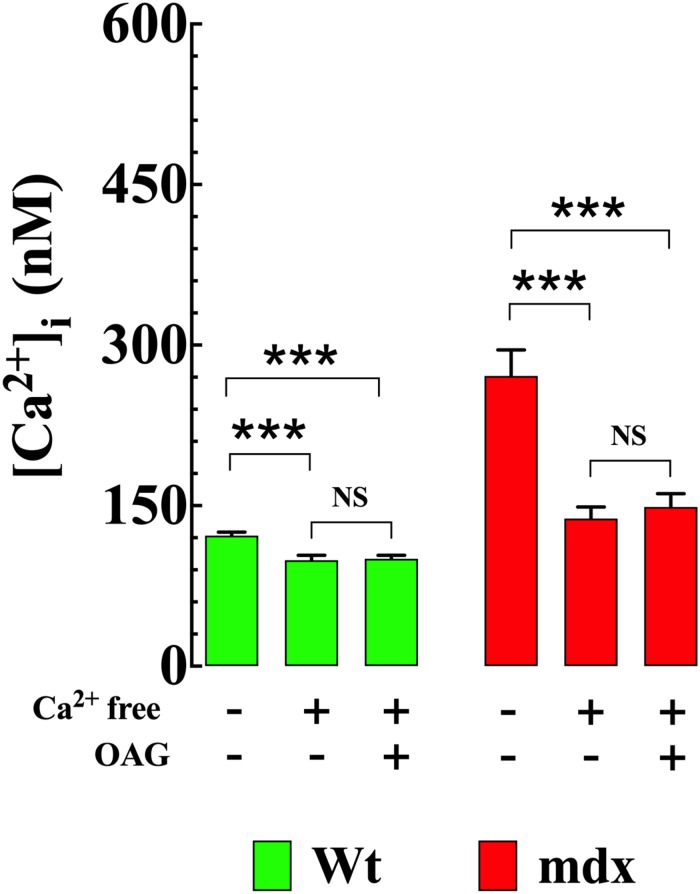
Removal of [Ca^2+^]_*e*_ prevents the OAG-induced elevation in [Ca^2+^]_i_. Removal of extracellular Ca^2+^ significantly lowered the [Ca^2+^]_i_ and provoked the inhibition of OAG (100 μM) induced elevation of [Ca^2+^]_i_ in VSMc isolated from Wt and mdx mice. On the horizontal axis are indicated the experimental conditions used to measure [Ca^2+^]_i_. For [Ca^2+^]_i_ measurements*: n*_mice_ = 4/experimental condition, *n*_cell_ = 15–18/genotype. Values are expressed as means ± S.D. for each condition. One-way ANOVA with Tukey’s post-test, ***p ≤ 0.001.

To establish the impact of extracellular Ca^2+^ ([Ca^2+^]_*e*_) on OAG-induced elevation of [Ca^2+^]_i_, Wt and mdx VSMCs were incubated for 5 min in Ca^2+^-free solution before OAG (100 μM) treatment. Exposure to reduced [Ca^2+^]_*e*_ significantly lowered [Ca^2+^]_i_ in Wt and mdx VSMCs but had a greater effect in mdx (−49%, from 271 ± 24, *n* = 15 to 138 ± 11, *n* = 18, *p* < 0.001) than in Wt (−18%, from 122 ± 3 nM, *n* = 15 to 100 ± 5 nM, *n* = 17, *p* < 0.001) VSMCs (Figure 4). In the absence of [Ca^2+^]_*e*_, the previously observed rise in [Ca^2+^]_i_ elicited by OAG in Ca^2+^ replete buffer was completely inhibited in both Wt and mdx VSMCs (Figure 3). In addition to the decrease in [Ca^2+^]_i_, incubation in Ca^2+^-free solution leads to a reversible depolarization of cell membrane potential in both genotypes of about 4–6 mV despite the presence of 2 mM Mg^2+^ (Supplementary Figure S2).

### SAR7334 Reduced [Ca^2+^]_i_ and Abolished the Increases of [Ca^2+^]_i_ and [Na^+^]_i_ Elicited by OAG

**FIGURE 5:**
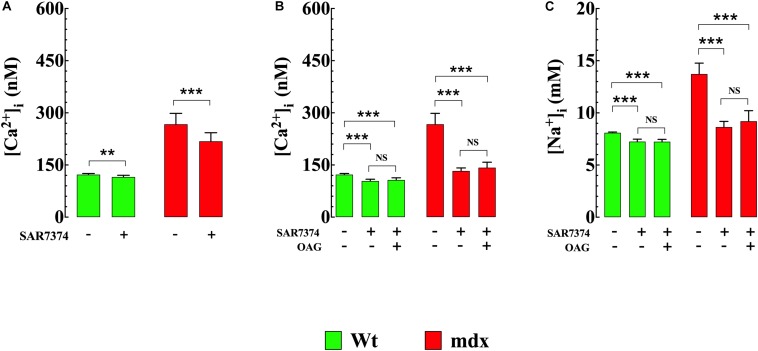
SAR7334 reduced [Ca^2+^]_i_ and [Na^+^]_i_ and blocked the elevation of [Ca^2+^]_i_ and [Na^+^]_i_ induced by OAG. [Ca^2+^]_i_ was measured in VSMc isolated from Wt and mdx mice before and after incubation in SAR7374 (0.1 μM or 1 μM), as well as after the exposure with SAR7374 and OAG (100 μM). **(A)** shows that preincubation in SAR7374 (0.1 μM) reduced significantly [Ca^2+^]_i_ and [Na^+^]_i_ in both genotypes. **(B)** illustrates that SAR7374 (1 μM) further reduced [Ca^2+^]_i_ and [Na^+^]_i_ and prevented the increase in intracellular [Ca^2+^] and [Na^+^] induced by OAG. **(C)** shows that SAR7374 (1 μM) reduced [Na^+^]_i_ in control and mdx VSMc and prevents the elevation of [Na^+^]_i_ induced by OAG. Over the horizontal axis are indicated the experimental conditions used to measure [Ca^2+^]_i_ and [Na^+^]_i_. For [Ca^2+^]_i_: *n*_mice_ = 3/experimental condition, *n*_cell_ = 17–20/genotype. For [Na^+^]_i_: *n*_mice_ = 3/experimental condition, *n*_cell_ = 13–19/genotype. Values are expressed as means ± S.D. for each condition. One-way ANOVA with Tukey’s post-test, **p < 0.01, ***p ≤ 0.001.

**FIGURE 6:**
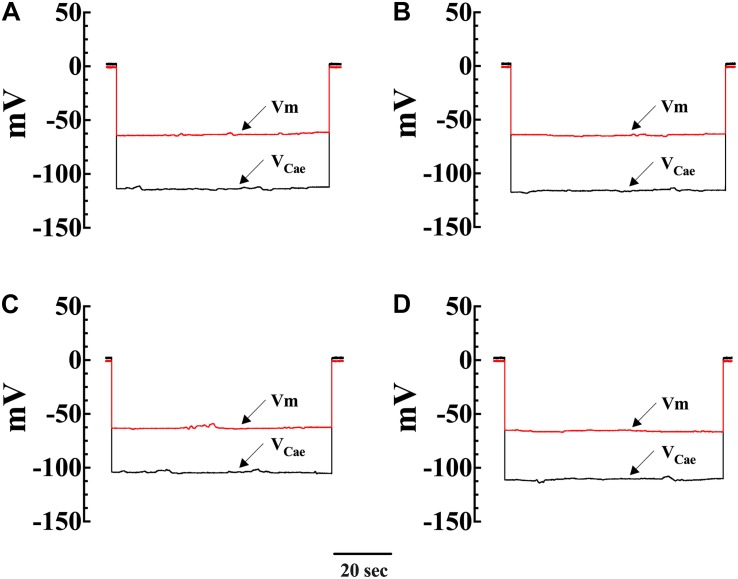
Effects of SAR7334 on [Ca^2+^]_i_ in Wt and mdx VSMC. A typical response obtained with a conventional microelectrode (Vm) and a Ca^2+^-selective microelectrode (V_Cae_) from a Wt and mdx VSMCs before and after SAR7334 (1 μM) treatment. **(A)** shows the recording from a Wt VSMC before SAR7334 treatment (Vm: –63 mV and [Ca^2+^]_i_ 124 nM) and **(B)** after SAR7334 incubation (Vm: –64 mV and [Ca^2+^]_i_ 98 nM). The Vm and [Ca^2+^]_i_ measurements show in **(A,B)** were carried in the same smooth muscle cell. **(C,D)** represent the determination of Vm and [Ca^2+^]_i_ before (Vm –63 mV and [Ca^2+^]_i_ 280 nM) and after SAR7334 application (Vm: –64 mV and [Ca^2+^]_i_ 147 nM) in a mdx VSMCs. The Vm and [Ca^2+^]_i_ measurements in show **(C,D)** were carried in the same smooth muscle cell.

To gain insight into molecular mechanisms resulting in [Ca^2+^]_i_ elevation upon exposure to OAG, we measured [Ca^2+^]_i_ in Wt and mdx VSMCs before and after incubation with SAR7334 which is a blocker of TRPC6 and TRPC3 channels (Maier et al., 2015), and then again after exposure to OAG (100 μM). Pretreatment with SAR7334 significantly lowered [Ca^2+^]_i_ in dose-dependent manner in both genotypes. Pretreatment with 0.1 μM SAR7334, a concentration that block mostly TRPC6 channels (Maier et al., 2015) reduced [Ca^2+^]_i_ in Wt by 6% and in mdx VSMCs by18% (Figure 5A). Preincubation with SAR7334 (1 μM) that blocks TRPC3 and 6 channels (Maier et al., 2015) provoked further reduction of [Ca^2+^]_i_ in Wt (15%) and mdx VSMCs (50%) (Figure 5B). The Figure 6 shows representative records of the effects of SAR7334 on [Ca^2+^]_i_ in Wt VSMCs (Figure 6B), and mdx VSMCs (Figure 6D). In addition, SAR7334 1 μM also reduced [Na^+^]_i_ in Wt (13%) and in mdx VSMCs (35%) (Figure 5C) and prevented any significant increase in [Ca^2+^]_i_ and [Na^+^]_i_ upon exposure to OAG in both genotypes (Figures 5B,C).

### Cyclic Stretch Provokes Larger Increase of [Ca^2+^]_i_ and [Na^+^]_i_ in mdx VSMCs

**FIGURE 7:**
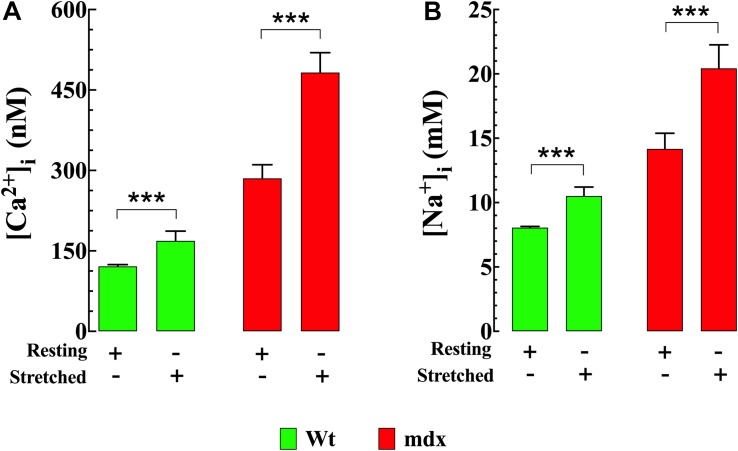
Stretch induces elevation of [Ca^2+^]_i_ and [Na^+^]_i_ in VSMCs. Repetitive mechanical (30 cycles/min) elongation (20% of resting length) produced a significant elevation of [Ca^2+^]_i_ and [Na^+^]_i_ in Wt and mdx VSMc, however, the magnitude of the increase of intracellular Ca^2+^ and Na^+^ concentrations was greater in mdx than Wt **(A,B)**. For [Ca^2+^]_i_: *n*_mice_ = 4/experimental condition, *n*_cell_ = 20–28/genotype. For [Na^+^]_i_: *n*_mice_ = 3/experimental condition, *n*_cell_ = 10–19/genotype. Values are expressed as means ± S.D. for each condition. Student’s *t*-test, ***p ≤ 0.001.

**FIGURE 8:**
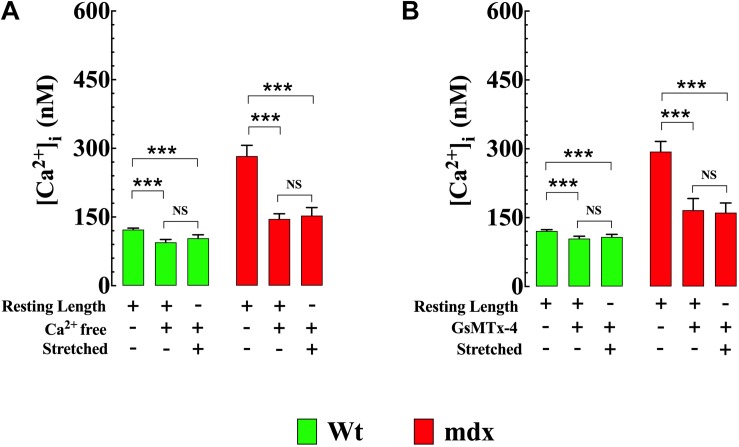
Reducing Ca^2+^ influx inhibits stretch-induced elevation of [Ca^2+^]_i_ in VSMCs. Removal of extracellular [Ca^2+^] **(A)** or blocking the mechanosensitive channels using GsMTx4 (5 μM) **(B)** reduced [Ca^2+^]_i_ and abolished the increase in [Ca^2+^]_i_ in response to stretch (20% of resting length) in both genotypes. For [Ca^2+^]_i_: *n*_mice_ = 5/experimental condition, *n*_cell_ = 13–19/genotype. Values are expressed as means ± S.D. for each condition. Student’s *t*-test, ***p ≤ 0.001.

Stretching smooth muscle cells has previously been shown to increase [Ca^2+^]_i_ (Zou et al., 2002; Ducret et al., 2010). Numerous members of the TRPC channel family, especially TRPC1, TRPC3, and TRPC6 are considered to be mechanosensitive channels (Friedrich et al., 2012; Takahashi et al., 2013) and are therefore possible candidates for this increase. In our VSMC stretch experiments [Ca^2+^]_i_ and [Na^+^]_i_ increased in both genotypes; however, the magnitude of the increases in Ca^2+^ and Na^+^ were greater in mdx than Wt. In Wt VSMCs [Ca^2+^]_i_ was elevated by 39% from 121 ± 3 nM, *n* = 25 to 169 ± 18 nM, *n* = 23 (*p* < 0.001) (Figure 7A) and [Na^+^]_i_ by 31% from to 8 ± 0.1 mM, *n* = 10 to 11 ± 1 mM, *n* = 10 (*p* < 0.001) (Figures 7B). In mdx VSMCs [Ca^2+^]_i_ was elevated by 69% from 285 ± 25 nM, *n* = 25 to 482 ± 37 nM, *n* = 20 (*p* < 0.001) (Figure 7A) and [Na^+^]_i_ by 43% from to 14 ± 1.2 mM, *n* = 19 to 20 ± 1.8 mM, *n* = 12 (*p* < 0.001) (Figure 7B). To test whether the elevation of [Ca^2+^]_i_ associated with stretch was mediated by Ca^2+^ influx through sarcolemma Ca^2+^ channels, extracellular Ca^2+^ was removed and 2 mM MgCl_2_ and 1 mM EGTA were added to the bathing supernatant (see section “Materials and Methods”). Under these conditions the increase in [Ca^2+^]_i_ in response to stretch was abolished in both Wt and mdx VSMCs (Figure 8A). Re-addition of extracellular Ca^2+^ before repeating the stretch protocol allowed recovery of the increase in both genotypes. These results suggest that a Ca^2+^ influx was involved in the elevation of [Ca^2+^]_i_ upon the mechanical stretch. To further dissect the mechanism involved in the stretch-induced elevation of [Ca^2+^]_i_ in VSMCs we tested the effect of GsMTx-4 (5 μM), which is known to inhibit mechanosensitive channels (Spassova et al., 2006; Bowman et al., 2007). GsMTx-4 completely inhibited the stretch-induced increases of [Ca^2+^]_i_ in both genotypes (Figure 8B). Additionally, we examined whether the stretch-induced elevation of [Ca^2+^]_i_ was mediated via L-type voltage-gated Ca^2+^ channels by incubating the VSMCs with the Ca^2+^ channel blocker nifedipine (10 μM). The stretch-induced increase in [Ca^2+^] was not modified by nifedipine in either genotype (Supplementary Figure S3).

### Cyclic Stretch Provokes Cell Damage in mdx VSMCs

**FIGURE 9:**
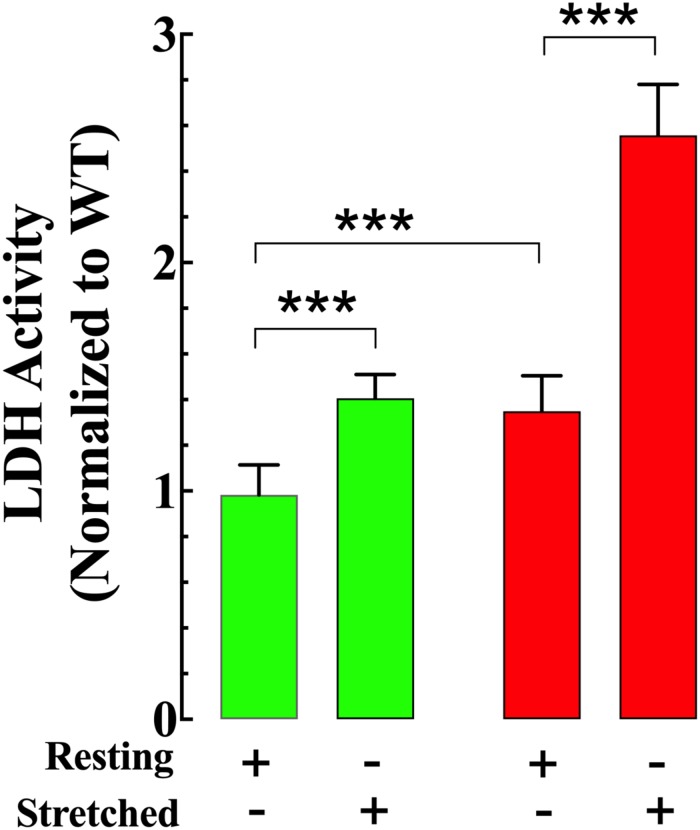
Stretch induces greater cell damage in mdx VSMCs. LDH activity in the supernatant from non-stretch VSMCs was higher in mdx than Wt. Muscle elongation increases LDH activity in both genotypes; however, it was much greater in mdx than Wt. For LDL: *n*_mice_ = 3/experimental condition, *n*_cell_ = 17–20/genotype. Values are expressed as means ± S.D. for each condition. Student’s *t*-test, ***p ≤ 0.001.

Resting LDH activity in the supernatant from non-stretched mdx VSMCs (a marker of cell damage) was 35% greater than in the supernatant from Wt VSMCs (Figure 9). Muscle stretching increased LDH activity in both genotypes; however, the increase was more significant in mdx than Wt VSMCs (41% in Wt vs. 90% in mdx VSMCs) (Figure 9).

### Measurements of Protein Expression

**FIGURE 10:**
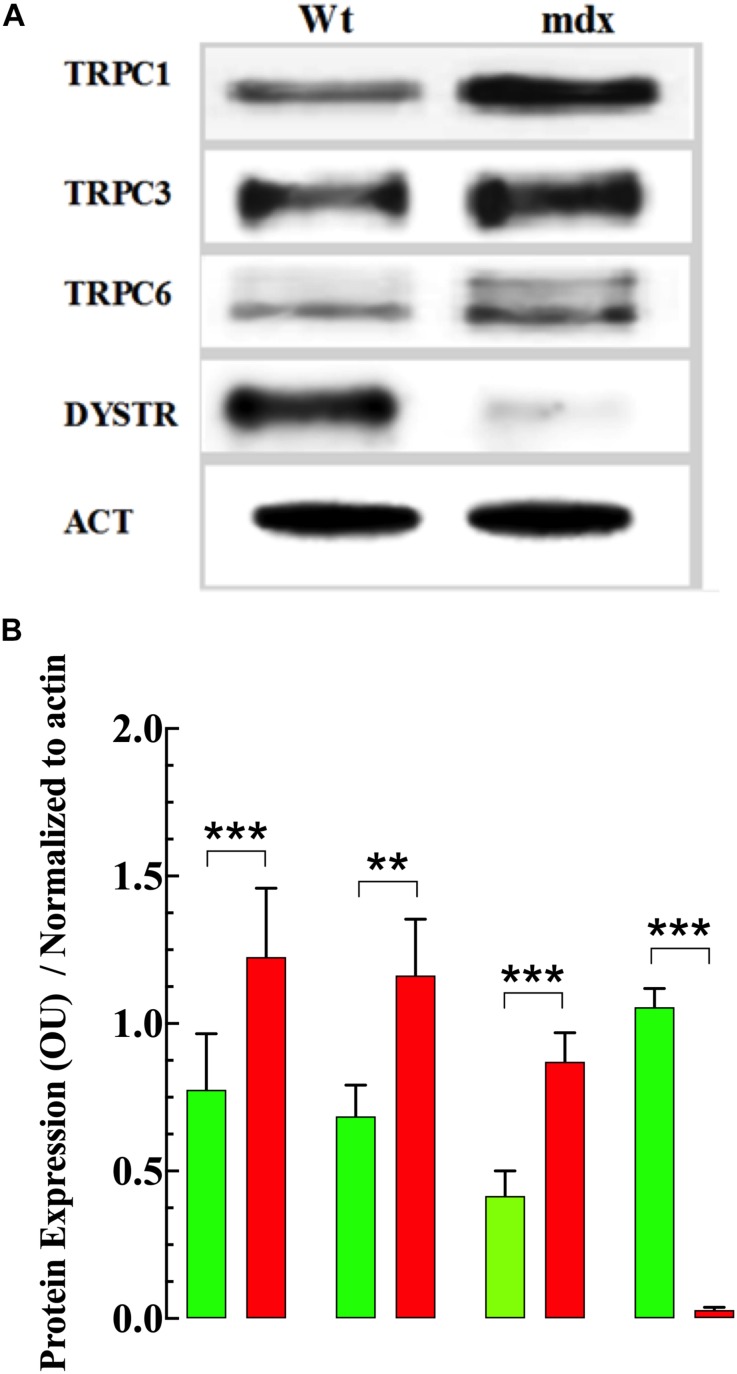
Expression of TRPC isoforms. Figure 10. Expression of TRPC isoforms. Representative fluorescent Western blot analysis of the expression of TRPC1, TRPC3, TRPC6, and dystrophin (DYSTR) proteins in Wt and mdx VSMCs **(A)**. Densitometric analysis of individual experiment fluorescent Western blots shown in **(B)**. Data were normalized to actin and expressed as mean optical unit values ± S.D. *n*_mice_ = 3. Paired *t*-test, **p ≤ 0.01, ***p ≤ 0.001.

To determine whether the elevation of [Ca^2+^]_i_ and [Na^+^]_i_ and enhanced response to OAG observed in dystrophic VSMs was associated with changes in TRPC protein in the membrane, the expression of TRPC1, -3 and -6 were measured using Western blot analysis. Analysis of these blots demonstrated that TRPC1, TRPC3, and TRPC6 were significantly upregulated in VSMCs from mdx compared to Wt mice (Figures 10A,B and Supplementary Figure S4).

## Discussion

To the best of our knowledge, this is the first comprehensive study of [Ca^2+^]_i_ and [Na^+^]_i_ dysregulation in mdx VSMCs. The main findings in the present study are the following: (i) quiescent VSMCs isolated from mdx mice have [Ca^2+^]_i_ and [Na^+^]_i_ overload; (ii) the increase in [Ca^2+^]_i_ and [Na^+^]_i_ induced by exposure to OAG was greater in mdx than Wt VSMCs; (iii) Removal of extracellular Ca^2+^ or blockade of TRPC3 and -6 channels abolished the increases of [Ca^2+^]_i_ and [Na^+^]_i_ elicited by OAG; (iv) Muscle stretch-induced elevation of [Ca^2+^]_i_, and [Na^+^]_i_ was significantly higher in mdx than Wt VSMCs and removal of extracellular Ca^2+^ or exposure to mechanosensitive channel blockers inhibited the increase in [Ca^2+^]_i_ linked to mechanical stretch in both mdx and Wt VSMCs; (v) Baseline and stretch-induced LDH leak was significantly higher in mdx than Wt VSMCs; (vi) Expression of TRPC1, -3, and -6 proteins was upregulated in mdx compared to Wt VSMCs.

Duchenne muscular dystrophy is a lethal muscle disease characterized by the absence of dystrophin, which leads to progressive membrane injury and subsequent changes in intracellular Ca^2+^ homeostasis and cellular dead (Ervasti et al., 1990). Dystrophin is the major component dystrophin-glycoprotein complex, which allows the interaction between the cytoskeleton and the and extracellular matrix (Ervasti and Campbell, 1993). Dystrophin is also present in the smooth muscle, playing a similar role than in skeletal muscle (North et al., 1993; Sharma et al., 2008). Deficiency of dystrophin in striated muscle cells results in alterations intracellular ion dyshomeostasis and muscle degeneration (Lopez et al., 1987; Danialou et al., 2001; Allen and Whitehead, 2011; Altamirano et al., 2012, 2014). In smooth muscle, the lack of dystrophin has been related with different alterations in the respiratory, gastrointestinal tract and the vascular bed (Miike et al., 1987; Barohn et al., 1988; Jaffe et al., 1990; Sun, 2015; Brown et al., 2018).

Intracellular calcium plays an essential role under physiological conditions to regulate many different processes in VSMCs (Berridge et al., 2003; Huang et al., 2018). Quiescent and healthy excitable cells maintain an [Ca^2+^]_i_ in the vicinity of 100–120 nM versus an extracellular [Ca^2+^] of 1.8 mM (Lopez et al., 1983; Mijares et al., 2014; Lopez et al., 2018). The activity of membrane ion channels, plasma membrane ATP-dependent Ca^2+^-pump, Na^+^/Ca^2+^ exchangers, and an endoplasmic reticulum Ca^2+^ ATPase preserve the concentration gradient (Karaki et al., 1997). Our data show that quiescent VSMCs isolated from mdx mice have an intracellular Ca^2+^ and Na^+^ overload compared with non-dystrophic Wt VSMCs. A substantial increase in [Ca^2+^]_i_ has been reported in intact skeletal muscle from DMD patients and an altered intracellular Ca^2+^ and Na^+^ homeostasis has been observed in the skeletal and cardiac muscle cells from mdx mice (Altamirano et al., 2014; Lopez et al., 2017, 2018). Chronic elevation in [Ca^2+^]_i_ may activate hydrolytic enzymes (proteases, nucleases, and lipases), and subsequently compromise energy production, intracellular ion regulation, ROS production and ultimately result in cell death (Nicotera and Orrenius, 1998; Ascah et al., 2011; Altamirano et al., 2013, 2014; Lopez et al., 2017). Prevention of chronic elevation of [Ca^2+^]_i_ may exert a myoprotective effect on mdx VSMCs precluding cell death.

The TRPC channels are expressed in vascular smooth muscle vessels playing diverse physiological cellular responses (Yip et al., 2004; Inoue et al., 2006). We have demonstrated that application of OAG, a membrane-permeable diacylglycerol analog which activates TRPC3 and TRPC6 channels (Hofmann et al., 1999; Liu et al., 2005) produced a robust elevation of [Ca^2+^]_i_ and [Na^+^]_i_ in Wt and mdx, however, the increment was more significant in mdx than Wt VSMCs. Western blots showed a significant upregulation of TRPC1 -3, -6 proteins in mdx VSMCs compare to age-matched Wt, which probably contribute to the observed intracellular Ca^2+^, and Na^+^ overload and also to the greater responsiveness to OAG found in mdx VSMCs.

Ca^2+^-free solution inhibited the observed rise in [Ca^2+^]_i_ induced by OAG and induced a reversible depolarization of cell membrane potential. The effect of removing extracellular Ca^2+^ on resting membrane potential in smooth muscle has been previously reported by other groups (Bulbring and Tomita, 1970; Kuriyama and Tomita, 1970). Furthermore, the incubation of VSCMs with SAR7334, a TRPC3 and TRPC6 blocker (Maier et al., 2015), reduced [Ca^2+^]_i_ in a dose-dependent manner and also blocked the increases in [Ca^2+^]_i_ and [Na^+^]_i_ elicited by OAG in both Wt and mdx VSMCs. Based on SAR7334 pharmacological dose blocking effect on TRPC3 and TRPC6 channels (Maier et al., 2015), we can speculate that the contribution of TRPC3 channels to the VSMCs intracellular Ca^2+^ dyshomeostasis is more significant than TRPC6 channels. The increase of [Ca^2+^]_i_ induced by OAG was not affected by the Ca^2+^ channel inhibitor nifedipine, which suggests that the activation of L-type Ca^2+^ channels is not part of the mechanism by which OAG induced elevation of [Ca^2+^]_i_ and [Na^+^]_i_ in VSMCs. Dysregulation of TRPC channels has been associated with diverse vascular pathologies (Mandegar et al., 2002; Yu et al., 2004; Kumar et al., 2006) which could explain, at least in part, the severe pulmonary and systemic hypertension reported in children and adolescents suffering from DMD (Yotsukura et al., 1988; Braat et al., 2015).

Previous studies have demonstrated that VSCM stretch induces elevation of [Ca^2+^]_i_ (Zou et al., 2002; Ducret et al., 2010). TRPC channels, especially TRPC1, TRPC3, and TRPC6, are considered as mechanosensitive channels (Friedrich et al., 2012; Takahashi et al., 2013). Furthermore, the Gq/11 protein has been recognized as mechanosensors involve in the myogenic vasoconstriction in VSMCs of small resistance arteries (Mederos et al., 2008, 2016). Gq/11-coupled receptors appear to be linked to the TRPC channels provoking the activation of TRPC channels in a G protein-dependent manner (Mederos et al., 2008). Here, we have shown evidence that a Ca^2+^ influx pathway activated by mechanosensors in VSMCs appears to be mediated by canonical cationic channel, which seems to be more critical in mdx VSMCs than Wt. Extracellular Ca^2+^ influx mediated by the voltage-dependent L-type Ca^2+^ channels has been suggested to intervene in VSMC stretch-mediated activation (Murase et al., 2001; Park et al., 2003; Ito et al., 2008). However, the fact that nifedipine did not inhibit stretch-induced [Ca^2+^]_i_ elevation does not support this hypothesis.

Basal LDH activity in the extracellular medium was higher in the supernatant from mdx compared to Wt VSMCs, which is consistent with the idea that the absence of dystrophin leads to chronic injury due to a lack of structural support at the sarcolemma. Besides, stretching further increased extracellular LDH activity, an indicator that this muscle stretch yielded some degree of cell injury in both genotypes. However, because the increase was higher in mdx than Wt, these data support the view that the lack of dystrophin makes VSCMs more sensitive to contraction-induced damage (Petrof et al., 1993; Grady et al., 1997; Brooks, 1998). The intracellular Ca^2+^ elevation after VSMCs stretch was suppressed entirely in both genotypes by removal of extracellular Ca^2+^ or GsMTx-4, indicating that this event is mediated by Ca^2+^ influx from the extracellular side which appears to be through a GsMTx-4 sensitive pathway.

### Study Limitations

Despite the novelty of our study, some limitations should be acknowledged. First, we used a pharmacological approach to characterize the involvement of TRPC channels in the dysregulation of intracellular Ca^2+^ observed in VSMCs from mdx mice, and we did not study the functional aspects of TRPC channels. Secondly, we did not carry out experiments in which TRPC1, -3, and -6 channels were individually or collectively downregulated using siRNA. Therefore, we were unable to assess whether decreasing TRPC channel expression rescues or improves intracellular Ca^2+^ regulation in mdx VSMCs.

## Conclusion

**FIGURE 11:**
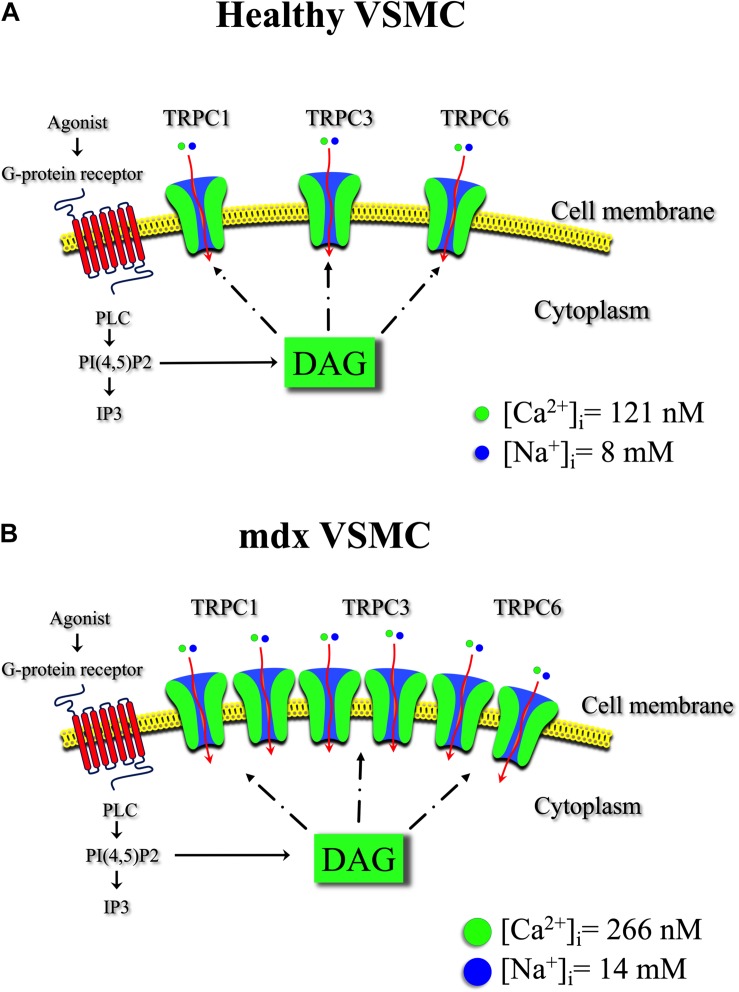
Transient receptor potential canonical channel in healthy and mdx VSMCs. Schematic representation of TRPC channels isoforms and the effect of diacylglycerol (DAG) on intracellular [Ca^2 +^ ] and [Na^+^] in healthy **(A)** and in mdx VSMC **(B)**. Binding of the agonist to the G-protein-coupled receptor leads to phospholipase C (PLC) activation. The activation of PLC hydrolyzes phosphatidyl 4-5 biphosphate PI(4,5)P2 to produce diacylglycerol (DAG) and IP3. DAG contributes to TRPC3 and TRPC6 channels, but also TRPC1 activation under heteromeric complexes allowing Ca^2+^ and Na^+^ influx into the vascular muscle cell. A chronic increase in Ca^2+^ and Na^+^ flux through the upregulated TRPC channels in mdx VSMCs contribute to the observed elevate [Ca^2+^]_i_ and [Na^+^]_i_.

This study provides direct evidence of anomalous regulation of resting intracellular Ca^2+^ and Na^+^ in VSMCs from mdx mice. The imbalance of [Ca^2+^]_i_ and [Na^+^]_i_ appears to be mediated mostly through TRPC channels since their pharmacological blocking activity markedly protected mdx VSMCs (Figure 11). Further, we have demonstrated the presence of an abnormal stretch-induced elevation of [Ca^2+^]_i_ in mdx VSMCs, which also appears to be mediated by TRPC channels. The originality of our paper stands in revealing the relevance of TRPC channels in the pathology of VSMCs in mdx mice. TRPC channels could be promising targets to help manage symptoms and slow the progression of this devastating disease.

## Data Availability Statement

The raw data supporting the conclusions of this article will be made available by the authors, without undue reservation, to any qualified researcher.

## Ethics Statement

All protocols used in the study were performed following the recommendations in the Guide for the Care and Use of Laboratory Animals of the National Institutes of Health and approved by the IACUC of Mount Sinai Medical.

## Author Contributions

JL and JA contributed to the conception and design of the study. JL, AU, GF, and EE performed the experiments. All authors contributed to the manuscript revision, read and approved the submitted version.

## Conflict of Interest

The authors declare that the research was conducted in the absence of any commercial or financial relationships that could be construed as a potential conflict of interest.

## Supplementary Material

FIGURE S1 Nifedipine does not block the OAG-induced increase in [Ca^2+^] in VSMCs. Incubation with nifedipine (NIFE) 10 μM did not abolish the increase in [Ca^2+^]_i_ induced by OAG 100 μM in VSMCs. For [Ca^2+^]_i_: *n*_mice_ = 3/experimental condition, *n*_cell_ = 10–17/genotype. Values are expressed as means ± S.D. for each condition. Student’s *t*-test, ****p* ≤ 0.001.

FIGURE S2 Effects of removal of extracellular Ca2 + on the resting membrane potential. The omission of the Ca^2+^ from the extracellular media induced a partial membrane depolarization (4–6 mV) in both genotypes. The reintroduction of Ca^2+^ to the bathing media reverses the observed depolarization. For Vm: *n*_mice_ = 3/experimental condition, *n*_cell_ = 20–26/genotype. Values are expressed as means ± S.D. for each condition. Student’s *t*-test, ****p* ≤ 0.001.

FIGURE S3 Stretching induced elevation of [Ca^2+^]_i_ in VSMCs is not inhibited by nifedipine. Exposure with nifedipine (NIFE) 10 μM did not prevent the stretch (20% of resting length) induces elevation of [Ca^2+^]_i_ in VSMCs. For [Ca^2+^]_i_: *n*_mice_ = 3/experimental condition, *n*_cell_ = 9–14/genotype. Values are expressed as means ± S.D. for each condition. Student’s *t*-test, ****p* ≤ 0.001.

FIGURE S4 TRPC and Dystrophin protein levels in VSMCs. Each panel shows representative TRPC1, TRPC3, TRPC6 and Dystrophin protein expressions using corresponding fluorescent antibody. Data are presented as optical unit (OU) values normalized to Actin signal. Left *Y* axis shows MW sizes (kDa) of corresponding protein standard size markers. Right *Y* axis is labeled with name and size (kDa) of corresponding protein signal on the representative blot. Top *X* axis contains names of total protein extract samples loaded onto the representative gel.
