# Supplementary figures and images for: Contribution of TRPC Channels to Intracellular Ca2 + Dyshomeostasis in Smooth Muscle From mdx Mice
Source: Front Physiol. 2020 Feb 20;11:126. doi: 10.3389/fphys.2020.00126 (PMC7044154; doi:10.3389/fphys.2020.00126)

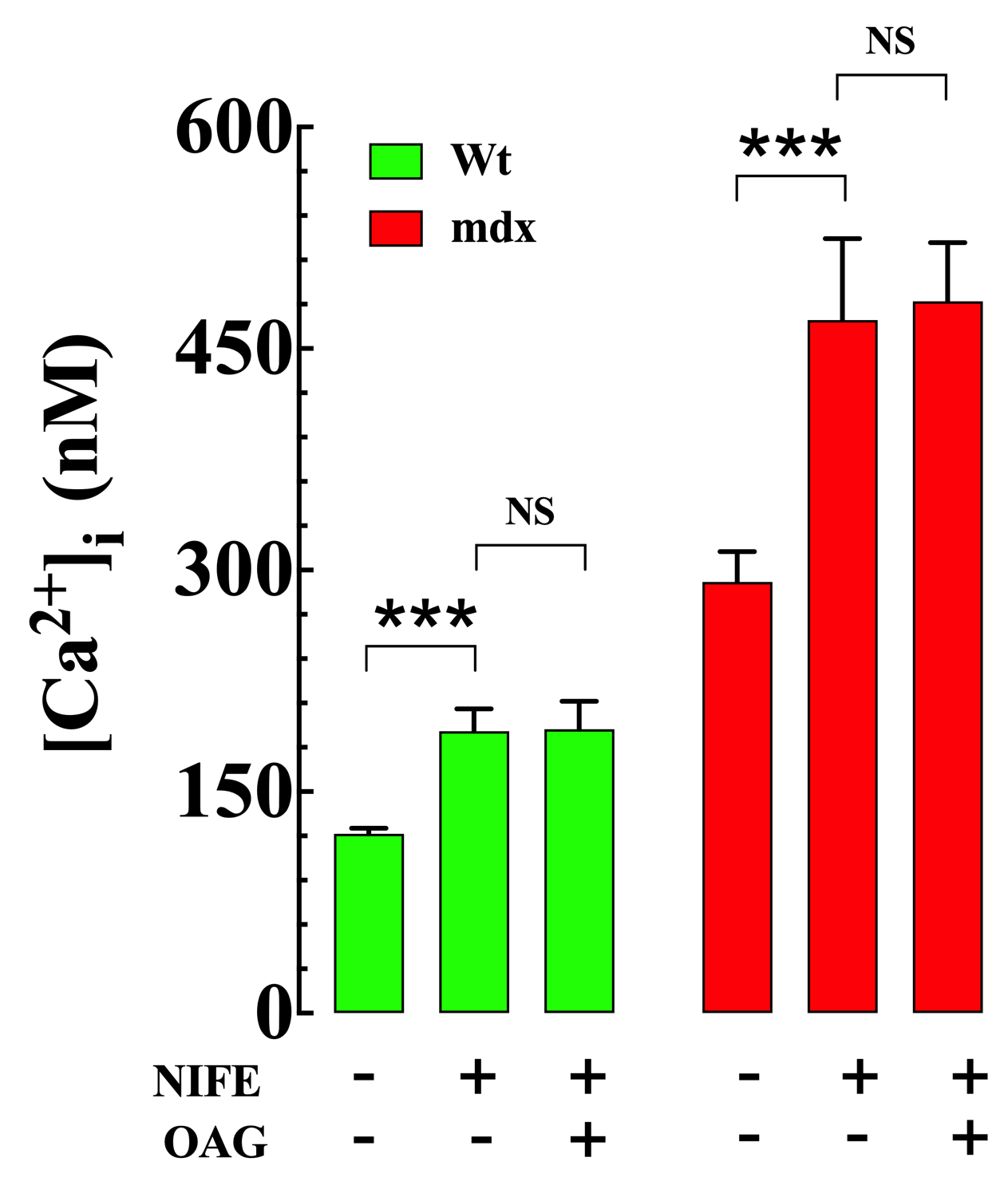

Supplement: FIGURE S1 — Nifedipine does not block the OAG-induced increase in [Ca2+] in VSMCs. Incubation with nifedipine (NIFE) 10 μM did not abolish the increase in [Ca2+]i induced by OAG 100 μM in VSMCs. For [Ca2+]i: nmice = 3/experimental condition, ncell = 10–17/genotype. Values are expressed as means ± S.D. for each condition. Student’s t-test, ***p ≤ 0.001. [file Image_1.TIFF]

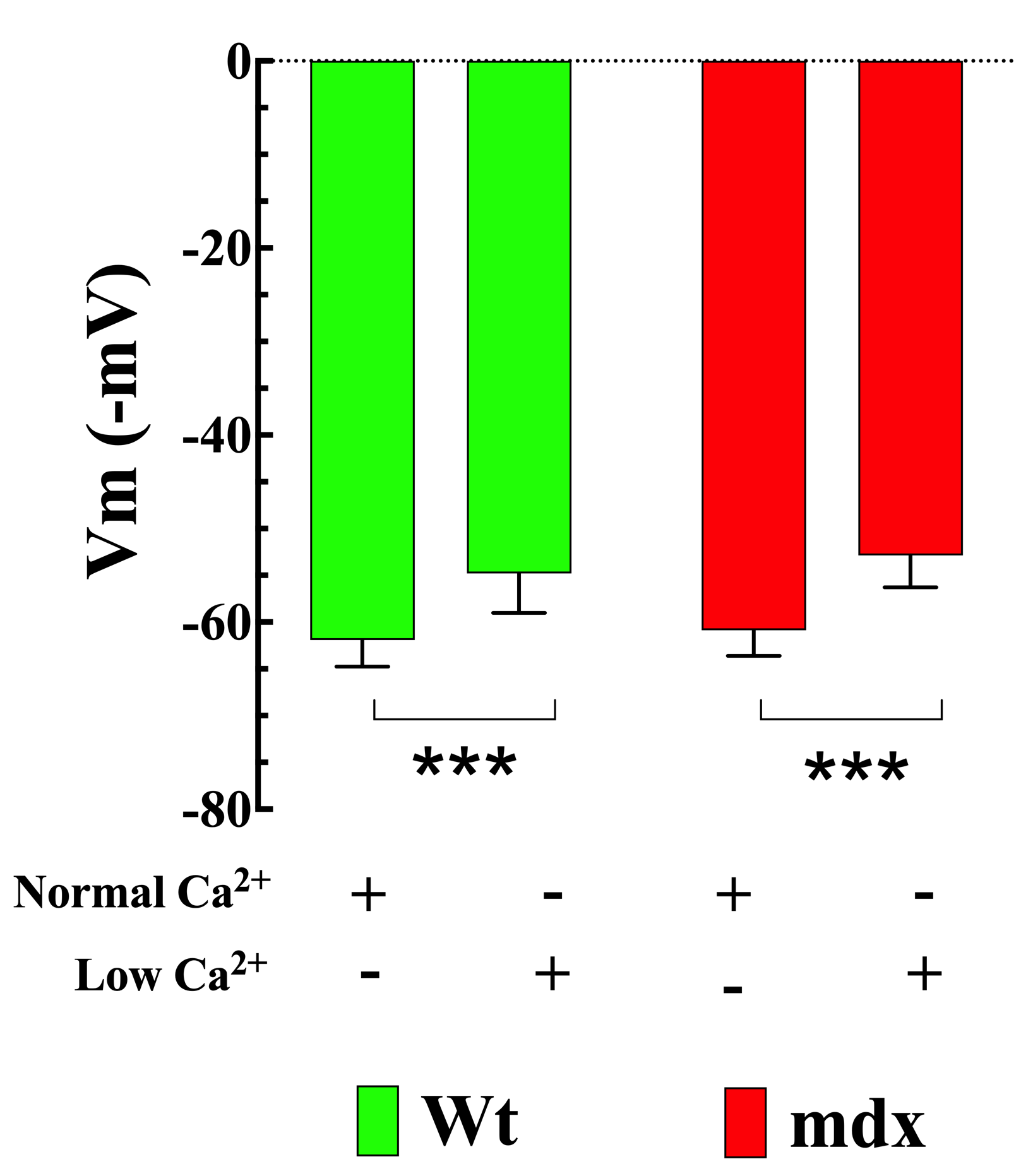

Supplement: FIGURE S2 — Effects of removal of extracellular Ca2 + on the resting membrane potential. The omission of the Ca2+ from the extracellular media induced a partial membrane depolarization (4–6 mV) in both genotypes. The reintroduction of Ca2+ to the bathing media reverses the observed depolarization. For Vm: nmice = 3/experimental condition, ncell = 20–26/genotype. Values are expressed as means ± S.D. for each condition. Student’s t-test, ***p ≤ 0.001. [file Image_2.TIFF]

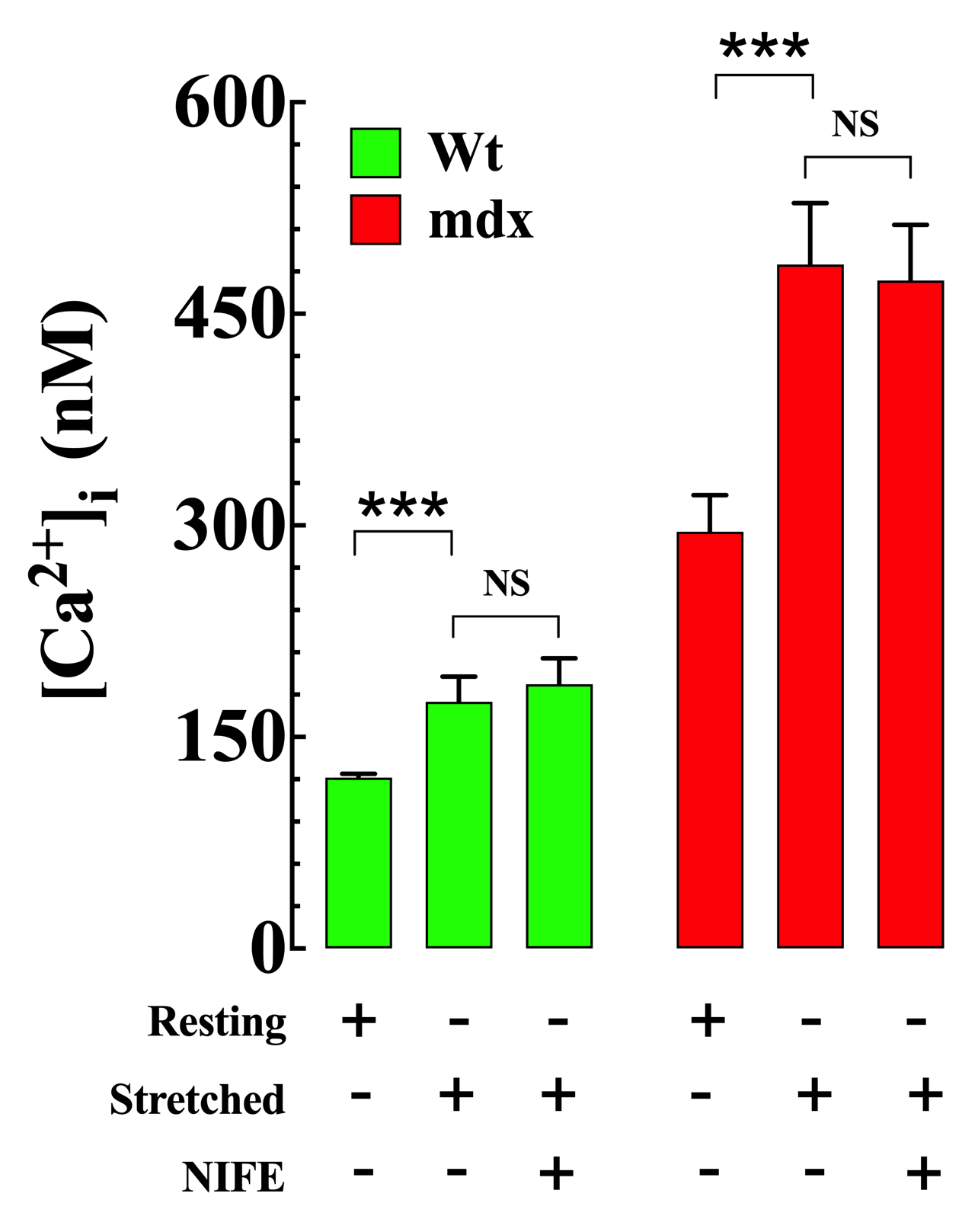

Supplement: FIGURE S3 — Stretching induced elevation of [Ca2+]i in VSMCs is not inhibited by nifedipine. Exposure with nifedipine (NIFE) 10 μM did not prevent the stretch (20% of resting length) induces elevation of [Ca2+]i in VSMCs. For [Ca2+]i: nmice = 3/experimental condition, ncell = 9–14/genotype. Values are expressed as means ± S.D. for each condition. Student’s t-test, ***p ≤ 0.001. [file Image_3.TIFF]

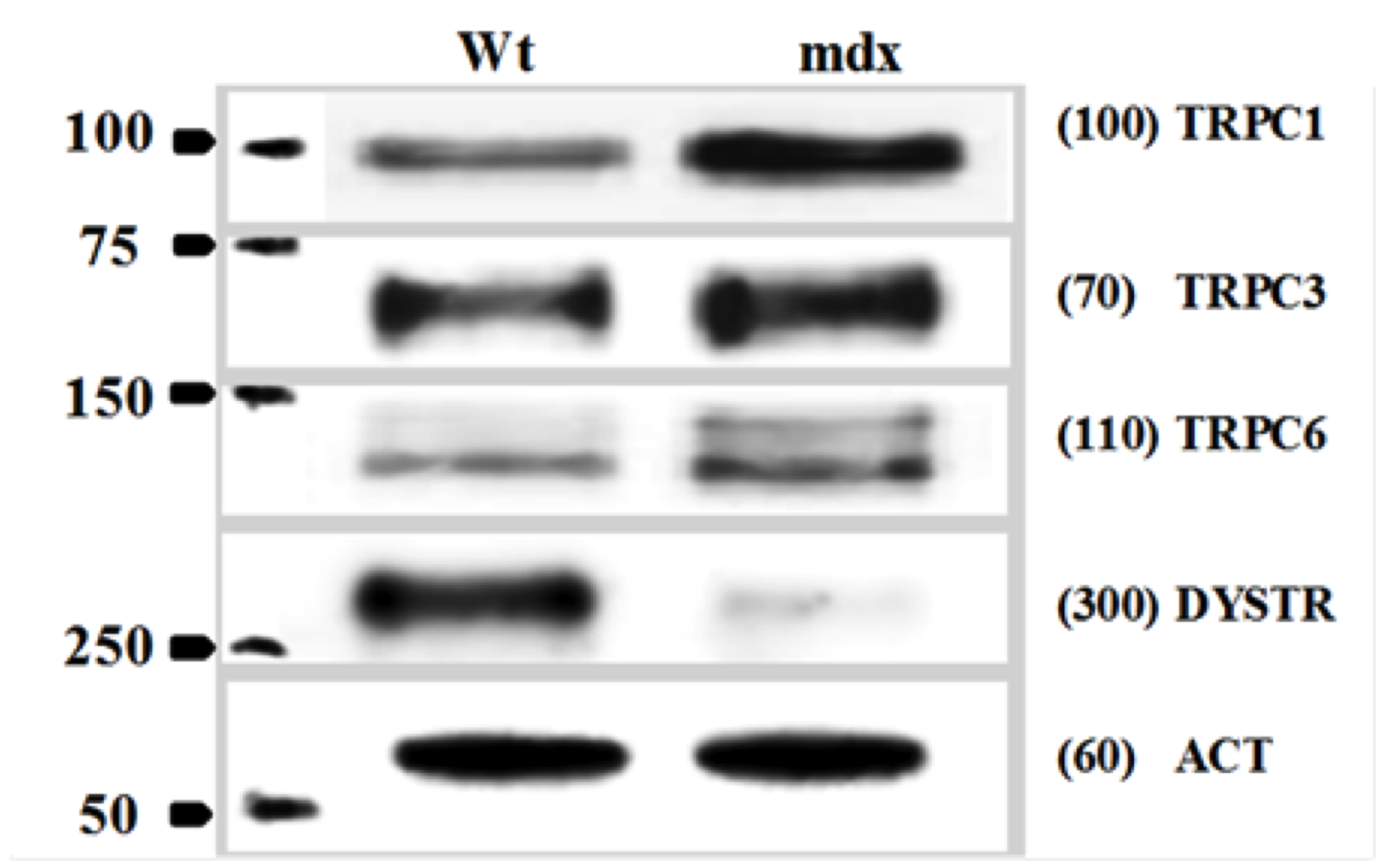

Supplement: FIGURE S4 — TRPC and Dystrophin protein levels in VSMCs. Each panel shows representative TRPC1, TRPC3, TRPC6 and Dystrophin protein expressions using corresponding fluorescent antibody. Data are presented as optical unit (OU) values normalized to Actin signal. Left Y axis shows MW sizes (kDa) of corresponding protein standard size markers. Right Y axis is labeled with name and size (kDa) of corresponding protein signal on the representative blot. Top X axis contains names of total protein extract samples loaded onto the representative gel. [file Image_4.TIFF]
